# Supplementary material for: Decision aids in patients with osteoporosis: A scoping review
Source: PLoS One. 2025 Jul 15;20(7):e0328230. doi: 10.1371/journal.pone.0328230 (PMC12262833; doi:10.1371/journal.pone.0328230)
Supplement: S1 File — (DOCX) [file pone.0328230.s001.docx]

**Preferred Reporting Items for Systematic reviews and Meta-Analyses extension for Scoping Reviews (PRISMA-ScR) Checklist**

| **SECTION** | **ITEM** | **PRISMA-ScR CHECKLIST ITEM** | **REPORTED ON PAGE #** |
| --- | --- | --- | --- |
| **TITLE** | | | |
| Title | 1 | Identify the report as a scoping review. | Decision Aids in Patients with Osteoporosis: A Scoping Review |
| **ABSTRACT** | | | |
| Structured summary | 2 | Provide a structured summary that includes (as applicable): background, objectives, eligibility criteria, sources of evidence, charting methods, results, and conclusions that relate to the review questions and objectives. | Purpose This scoping review described the use and effectiveness of decision aids in clinical decision-making among individuals with osteoporosis.  Methods PubMed, CINAHL, Web of Science, Embase, Cochrane Library, China Knowledge Network, Wanfang Database, and China Biomedical Literature Database were searched. The search timeframe was from the establishment of the database to June 30, 2024. The included literature was summarized and analyzed.  Results There were eighteen papers altogether. Decision aids were used to help patients with osteoporosis with diagnosis, therapy choices, prescription recommendations, primary prevention, and secondary prevention. A description of the risks, benefits, and drawbacks associated with the decision scenario, an explanation of the patient's values and care preferences, interactive decision communication support, and informational guidance pertaining to the patient's physical condition and the decision topic were among the content elements of decision aids. Decision process indicators, decision quality indicators, and feasibility indicators were examples of endpoint indicators.  Conclusion With strong efficacy and viability, decision aids greatly enhances patients' decision-making experience and decision quality. In order to provide patients with osteoporosis with high-quality decision-making support, it will be necessary to conduct large-scale, randomized controlled studies in the future with the goal of guaranteeing homogeneous interventions, expand the scope and meaning of the application of decision aids in osteoporosis, improve professional support during the decision-making process, create scientific and useful decision-making aids, and take specific actions. |
| **INTRODUCTION** | | | |
| Rationale | 3 | Describe the rationale for the review in the context of what is already known. Explain why the review questions/objectives lend themselves to a scoping review approach. | Osteoporosis, a prevalent skeletal disorder among the elderly population, poses significant challenges to healthy aging. However, patients frequently encounter difficulties in making informed treatment decisions. Decision aids serve as evidence-based tools designed to facilitate preference-sensitive decision-making by presenting treatment options and outcomes, and have been formally recommended by the UK National Institute for Health and Care Excellence guidelines. Nevertheless, substantial heterogeneity persists regarding the thematic focus, core components, outcome measures, and clinical impacts of decision aids in OP management. This scoping review systematically examines the application of decision aids in OP patients, with particular emphasis on their thematic content, structural elements, and effectiveness, thereby providing evidence-based recommendations to inform both clinical practice and future research directions. |
| Objectives | 4 | Provide an explicit statement of the questions and objectives being addressed with reference to their key elements (e.g., population or participants, concepts, and context) or other relevant key elements used to conceptualize the review questions and/or objectives. | Participants: patients meeting the diagnostic criteria for osteoporosis published by WHO with T-value ≤ -2.5 for bone density of the midshaft bone (lumbar vertebrae 1-4, femoral neck, or total hip) or bone density of the distal 1/3 of the radius ≤ -2.5.  Concept: decision-aided interventions for decision-making for patients with osteoporosis through decision aids.  Context：The place where decision aids are applied, such as communities, nursing institutions or hospitals. |
| **METHODS** | | | |
| Protocol and registration | 5 | Indicate whether a review protocol exists; state if and where it can be accessed (e.g., a Web address); and if available, provide registration information, including the registration number. | N/A |
| Eligibility criteria | 6 | Specify characteristics of the sources of evidence used as eligibility criteria (e.g., years considered, language, and publication status), and provide a rationale. | Inclusion criteria:  All studies published after 2010 in any language that focus on the development, evaluation, or qualitative assessment of decision aids related to osteoporosis management.  Exclusion criteria:  Studies involving non-osteoporotic populations;  Interventions not specifically designed as decision aids (e.g., general educational materials); |
| Information sources* | 7 | Describe all information sources in the search (e.g., databases with dates of coverage and contact with authors to identify additional sources), as well as the date the most recent search was executed. | Search PubMed, CINAHL, Web of Science, Embase, Cochrane Library, China Knowledge Network, Wanfang Database, and China Biomedical Literature Database. The timeframe for searching is from the build date to June 30, 2024. |
| Search | 8 | Present the full electronic search strategy for at least 1 database, including any limits used, such that it could be repeated. | #1 ("Osteoporosis"[MeSH Terms] OR "osteoporos*"[Title/Abstract] OR "post traumatic osteoporos*"[Title/Abstract] OR "senile osteoporos*"[Title/ Abstract] OR "age related bone loss"[Title/Abstract] OR "age related bone losses"[Title/Abstract] OR "age related osteoporos*"[Title/Abstract])  #2 ("Decision Support Techniques"[MeSH Terms] OR "decision aid*"[Title/Abstract] OR "decision support*"[Title/Abstract] OR "decision technolog*"[ Title/Abstract] OR "decision technique*"[Title/Abstract] OR "decision algorithm*"[Title/Abstract] OR "decision intervention*"[Title/ Abstract] OR "decision material"[Title/Abstract])  #3 #1 AND #2 |
| Selection of sources of evidence† | 9 | State the process for selecting sources of evidence (i.e., screening and eligibility) included in the scoping review. | All abstracts were independently reviewed by two different members of the research team. Any disagreements over inclusion were resolved through consensus and, where necessary, discussion with a third member of the review team. |
| Data charting process‡ | 10 | Describe the methods of charting data from the included sources of evidence (e.g., calibrated forms or forms that have been tested by the team before their use, and whether data charting was done independently or in duplicate) and any processes for obtaining and confirming data from investigators. | The data included general information such as authors, year of publication, country, study site, sample information, study type, intervention form, content elements, application scope, and outcome indicators. |
| Data items | 11 | List and define all variables for which data were sought and any assumptions and simplifications made. | Variables: Decision aids format, development methodology (e.g., IPDAS criteria), study design, and population characteristics. |
| Critical appraisal of individual sources of evidence§ | 12 | If done, provide a rationale for conducting a critical appraisal of included sources of evidence; describe the methods used and how this information was used in any data synthesis (if appropriate). | N/A |
| Synthesis of results | 13 | Describe the methods of handling and summarizing the data that were charted. | The evidence distribution was tabulated by decision aids application scope, intervention format, and outcome measures. |
| **RESULTS** | | | |
| Selection of sources of evidence | 14 | Give numbers of sources of evidence screened, assessed for eligibility, and included in the review, with reasons for exclusions at each stage, ideally using a flow diagram. | 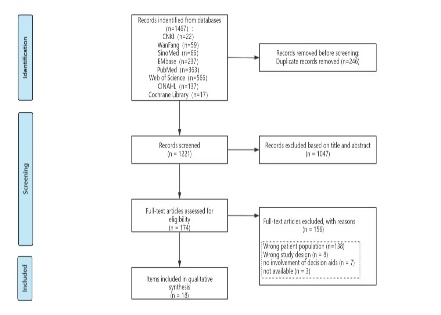 |
| Characteristics of sources of evidence | 15 | For each source of evidence, present characteristics for which data were charted and provide the citations. | The basic characteristics of the included literature are shown in Table 1. |
| Critical appraisal within sources of evidence | 16 | If done, present data on critical appraisal of included sources of evidence (see item 12). | N/A |
| Results of individual sources of evidence | 17 | For each included source of evidence, present the relevant data that were charted that relate to the review questions and objectives. | These are shown in Table 1. |
| Synthesis of results | 18 | Summarize and/or present the charting results as they relate to the review questions and objectives. | Decision aids support osteoporosis decisions across diagnosis, treatment, medication, and prevention levels. Their core components cover evidence presentation, risk-benefit comparisons, values clarification, and interactive support, with outcomes evaluating decision processes, quality, and feasibility. |
| **DISCUSSION** | | | |
| Summary of evidence | 19 | Summarize the main results (including an overview of concepts, themes, and types of evidence available), link to the review questions and objectives, and consider the relevance to key groups. | Decision aids can effectively enhance patients' knowledge base and engagement in decision-making while reducing decisional conflict and improving satisfaction. However, their application scope requires further expansion, and the current evidence regarding their effectiveness remains insufficient. |
| Limitations | 20 | Discuss the limitations of the scoping review process. | 1.Language bias: Only English-language studies were included.  2.Imaging restriction: Limited to DXA-based studies, excluding other modalities (e.g., MRI).  3.No quality assessment: Study methodological quality was not evaluated per scoping review guidelines. |
| Conclusions | 21 | Provide a general interpretation of the results with respect to the review questions and objectives, as well as potential implications and/or next steps. | Standardized development protocols for decision aids should be established, with future research prioritizing long-term outcome evaluation and the development of culturally adapted decision aids. |
| **FUNDING** | | | |
| Funding | 22 | Describe sources of funding for the included sources of evidence, as well as sources of funding for the scoping review. Describe the role of the funders of the scoping review. | This work was supported by the Basic Public Welfare Research Program of Zhejiang Province (Grant No. LTGY23H170005) and Zhejiang Medical and Health Science and Technology Project (Grant No. 2024KY644). |

JBI = Joanna Briggs Institute; PRISMA-ScR = Preferred Reporting Items for Systematic reviews and Meta-Analyses extension for Scoping Reviews.

* Where *sources of evidence* (see second footnote) are compiled from, such as bibliographic databases, social media platforms, and Web sites.

† A more inclusive/heterogeneous term used to account for the different types of evidence or data sources (e.g., quantitative and/or qualitative research, expert opinion, and policy documents) that may be eligible in a scoping review as opposed to only studies. This is not to be confused with *information sources* (see first footnote).

‡ The frameworks by Arksey and O’Malley (6) and Levac and colleagues (7) and the JBI guidance (4, 5) refer to the process of data extraction in a scoping review as data charting*.*

§ The process of systematically examining research evidence to assess its validity, results, and relevance before using it to inform a decision. This term is used for items 12 and 19 instead of "risk of bias" (which is more applicable to systematic reviews of interventions) to include and acknowledge the various sources of evidence that may be used in a scoping review (e.g., quantitative and/or qualitative research, expert opinion, and policy document).
