# Supplementary material for: Decision aids in patients with osteoporosis: A scoping review
Source: PLoS One. 2025 Jul 15;20(7):e0328230. doi: 10.1371/journal.pone.0328230 (PMC12262833; doi:10.1371/journal.pone.0328230)
Supplement: S2 File — (DOCX) [file pone.0328230.s002.docx]

**Supporting information 2**

**Scoping review data extraction stages.**

| No | Stage | Task | Reason/s |
| --- | --- | --- | --- |
| 1. | Stage 1 | Title and abstract screening | To ensure that the articles are eligible for full text screening |
| 2. | Stage 2 | Full text screening | Information about decision aids will be found in the methods and results section. |
| 3. | Final stage | To resolve conflicts | To ensure that articles that do not fully meet eligibility and the two independent reviewers do not agree to their inclusion, a third reviewer will be invited to resolve the conflicts. |
