# Supplementary material for: Decision aids in patients with osteoporosis: A scoping review
Source: PLoS One. 2025 Jul 15;20(7):e0328230. doi: 10.1371/journal.pone.0328230 (PMC12262833; doi:10.1371/journal.pone.0328230)
Supplement: S4 File — (DOCX) [file pone.0328230.s004.docx]

**Supporting information 2**

**Data extraction table template**

| Author | Year | Country | research site | study population | Study design | sample size | Forms of intervention | Content elements | Scope of Application | Outcome indicator |
| --- | --- | --- | --- | --- | --- | --- | --- | --- | --- | --- |
