# Supplementary material for: Decision aids in patients with osteoporosis: A scoping review
Source: PLoS One. 2025 Jul 15;20(7):e0328230. doi: 10.1371/journal.pone.0328230 (PMC12262833; doi:10.1371/journal.pone.0328230)
Supplement: S5 File — (DOCX) [file pone.0328230.s005.docx]

**Supporting information 1**

**Search strategy for PubMed on 30/6/2024**

| Search number | Query | Sort By | Filters | Search Details | Results | Time |
| --- | --- | --- | --- | --- | --- | --- |
| 1 | (((Osteoporosis) OR (post traumatic osteoporosis)) OR (senile osteoporosis)) OR (bone loss) | Most Recent |  | ("Osteoporosis"[MeSH Terms] OR "osteoporos*"[Title/Abstract] OR "post traumatic osteoporos*"[Title/Abstract] OR "senile osteoporos*"[Title/Abstract] OR "age related bone loss"[Title/Abstract] OR "age related bone losses"[Title/Abstract] OR "age related osteoporos*"[Title/Abstract]) | 112161 | 10:13:11 |
| 2 | (((Decision Support Techniques) OR (decision aid)) OR (decision support)) OR (decision technique) | Most Recent |  | ("Decision Support Techniques"[MeSH Terms] OR "decision aid*"[Title/Abstract] OR "decision support*"[Title/Abstract] OR "decision technolog*"[Title/Abstract] OR "decision technique*"[Title/Abstract] OR "decision algorithm*"[Title/Abstract] OR "decision intervention*"[Title/Abstract] OR "decision material"[Title/Abstract]) | 113402 | 10:14:30 |
| 3 | (((Osteoporosis) OR (post traumatic osteoporosis)) OR (senile osteoporosis)) OR (bone loss) AND (((Decision Support Techniques) OR (decision aid)) OR (decision support)) OR (decision technique) | Most Recent | English | ("Osteoporosis"[MeSH Terms] OR "osteoporos*"[Title/Abstract] OR "post traumatic osteoporos*"[Title/Abstract] OR "senile osteoporos*"[Title/Abstract] OR "age related bone loss"[Title/Abstract] OR "age related bone losses"[Title/Abstract] OR "age related osteoporos*"[Title/Abstract]) AND ("Decision Support Techniques"[MeSH Terms] OR "decision aid*"[Title/Abstract] OR "decision support*"[Title/Abstract] OR "decision technolog*"[Title/Abstract] OR "decision technique*"[Title/Abstract] OR "decision algorithm*"[Title/Abstract] OR "decision intervention*"[Title/Abstract] OR "decision material"[Title/Abstract]) | 363 | 10:14:35 |
